# Claudin-3 Overexpression Increases the Malignant Potential of Colorectal Cancer Cells: Roles of ERK1/2 and PI3K-Akt as Modulators of EGFR signaling

**DOI:** 10.1371/journal.pone.0074994

**Published:** 2013-09-19

**Authors:** Waldemir F. de Souza, Natalia Fortunato-Miranda, Bruno K. Robbs, Wallace M. de Araujo, Julio C. de-Freitas-Junior, Lilian G. Bastos, João P. B. Viola, José A. Morgado-Díaz

**Affiliations:** 1 Grupo de Biologia Estrutural, Programa de Biologia Celular, Centro de Pesquisas, Instituto Nacional de Câncer, Rio de Janeiro, Brazil; 2 Grupo de Regulação Gênica, Programa de Biologia Celular, Centro de Pesquisas, Instituto Nacional de Câncer, Rio de Janeiro, Brazil; Seoul National University, Republic of Korea

## Abstract

The altered expressions of claudin proteins have been reported during the tumorigenesis of colorectal cancer. However, the molecular mechanisms that regulate these events in this cancer type are poorly understood. Here, we report that epidermal growth factor (EGF) increases the expression of claudin-3 in human colorectal adenocarcinoma HT-29 cells. This increase was related to increased cell migration and the formation of anchorage-dependent and anchorage-independent colonies. We further showed that the ERK1/2 and PI3K-Akt pathways were involved in the regulation of these effects because specific pharmacological inhibition blocked these events. Genetic manipulation of claudin-1 and claudin-3 in HT-29 cells showed that the overexpression of claudin-1 resulted in decreased cell migration; however, migration was not altered in cells that overexpressed claudin-3. Furthermore, the overexpression of claudin-3, but not that of claudin-1, increased the tight junction-related paracellular flux of macromolecules. Additionally, an increased formation of anchorage-dependent and anchorage-independent colonies were observed in cells that overexpressed claudin-3, while no such changes were observed when claudin-1 was overexpressed. Finally, claudin-3 silencing alone despite induce increase proliferation, and the formation of anchoragedependent and -independent colonies, it was able to prevent the EGF-induced increased malignant potential. In conclusion, our results show a novel role for claudin-3 overexpression in promoting the malignant potential of colorectal cancer cells, which is potentially regulated by the EGF-activated ERK1/2 and PI3K-Akt pathways.

## Introduction

Tight junctions (TJs) are important structural components of the apical junctional complex in the epithelium, where they regulate various intracellular processes such as the establishment of apical-basal polarity and the flow of substances across the intercellular space [1]. Claudins are the main proteins that regulate the functions of TJs and are classified as a family of tetraspan integral membrane proteins, which currently comprises 27 members [2]. A myriad of diseases, including cancer, have been associated with alterations in the expression, stability and subcellular localization of claudin family members [3], [4], [5], [6]. However, the precise molecular mechanisms that regulate the expression and function of these proteins, particularly in colorectal cancer, are poorly understood.

The epidermal growth factor receptor (EGFR) is dimerized and activated by its extracellular ligand, EGF, which triggers a signaling cascade that leads to the activation of cytoplasmic pathways such as MAPK and PI3K-Akt [7], [8]; these pathways are known to modulate proliferation, differentiation and resistance to cell death [9], [10]. Studies have shown that these pathways are involved in events related to the carcinogenic processes in mouse epidermal and human gastric cancer cells [11], [12], as well as in the increased migratory and invasive potential during the epithelial-mesenchymal transition in human ovarian cells [13]. EGF-mediated signaling pathways are also known to play important roles in the organization of TJs, in which they regulate the expression and localization of claudin proteins. For instance, EGF was reported to induce the upregulation of claudins 1, 3 and 4, and the EGF-induced downregulation of claudin-2 increases the force of the intercellular barrier, as determined by an increased transepithelial electrical resistance (TER) in MDCK-II cells [14], [15]. However, using the same model (MDCK cells), other authors have reported that the downregulation of claudin-2 induced higher cell motility, even with increased TER [16]. Recently, the EGFR/ERK/c-Fos pathway was shown to up-regulate claudin-2, an increase that was correlated with increased intercellular permeability and cell migration in human lung adenocarcinoma cells [17], [18].

Little information is known about the molecular mechanisms underlying the alterations in claudin expression that are associated with colorectal tumorigenesis. We have shown that patients with colorectal cancer presented increased expression levels of claudins 1, 3 and 4, which altered the barrier function of TJs [19]. Recent studies have reported a controversial role for claudin-1 during colorectal carcinogenesis; increased claudin-1 expression was observed in hepatic metastatic lesions of colorectal cancer, but this expression was decreased in the lymph node metastases of colon carcinomas [20], [21]. Additionally, the ERK1/2 and PI3K pathways have been reported to mediate increases in EGF-induced claudin-2 expression in colon cancer cells; this event was accompanied by increases in proliferation, anchorage-independent growth and tumor growth *in vivo* [22]. Therefore, it is important to understand the molecular mechanisms that regulate the expression of other claudin family members and the implications of claudin overexpression in colorectal cancer progression.

In the present study, we show that the EGF-mediated increased expression of claudin-3 is related to increased cell migration and the formation of anchorage-dependent and anchorage-independent colonies in human colorectal adenocarcinoma HT-29 cells. Furthermore, we show that these events were mediated by the ERK1/2 and PI3K-Akt pathways. We also demonstrated that the forced overexpression of claudin-3 in HT-29 cells by genetic manipulation increased the malignant potential, while the overexpression of claudin-1 decreased cell migration. Most importantly, our results reveal that claudin-3 plays a role as tumor promoter when its expression is imbalanced and implicate the ERK1/2 and PI3K-Akt signaling pathways as modulators of claudin-3 upregulation-related tumor progression in colorectal cancer cells.

## Materials and Methods

### Materials

Anti-claudin-1 (Cat. no. 51–9000) and anti-claudin-3 (Cat. no. 34–1700) rabbit polyclonal antibodies as well as an anti-α-tubulin mouse monoclonal antibody (Cat. no. 32–2500) were obtained from Invitrogen Inc. (Carlsbad, CA, USA). Anti-Akt (Cat. no. 4691) and anti-p-Akt (Cat. no. 4058) rabbit monoclonal antibodies as well as an anti-ERK mouse monoclonal (Cat. no. 9107) antibody were purchased from Cell Signaling (Beverly, MA, USA). The anti-uvomorulin/E-cadherin rat monoclonal (Cat. no. U3254) and anti-p-ERK1/2 mouse monoclonal (Cat. no. M8159) antibodies were obtained from Sigma-Aldrich (St Louis, MO, USA). An anti-E-cadherin mouse monoclonal antibody (clone 36, Cat. no. 610182) was purchased from BD Biosciences (San Diego, CA, USA). Horseradish peroxidase-conjugated anti-rabbit and anti-mouse IgG were purchased from GE Healthcare (Chalfont St. Giles, UK). Alex-fluor-488 anti-rabbit (Cat. no. A11008) and Alex-fluor-546 anti-rat (Cat. no. A11010) were obtained from Molecular Probes (Eugene, Oregon, USA). The 2-(4-morpholinyl)-8-phenyl-1(4H)-benzopyran-4-one LY294002 (PI3K inhibitor) (Cat. no. L9908) was obtained from Sigma-Aldrich, the 2-(2-amino-3-methoxyphenyl)-4H-1-benzopyran-4-one PD98059 (MEK1 inhibitor) (Cat. no. 9900) was obtained from Cell Signaling, and EGF (Cat. no. PHG0311) was purchased from Invitrogen Inc.

### Cell Culture

The human colorectal adenocarcinoma cell lines Caco-2 (Cat. no. HTB-37) and HT-29 (Cat. no. HTB-38) as well as the human embryonic ***kidney*** cell line HEK-293 (Cat. no. CRL-1573) were obtained from the American Type Culture Collection (Manassas, VA, USA). Caco-2 cells present with a differentiated phenotype at the monolayer stage and possess a low invasive and metastatic potential [23], [24], [25], while the HT-29 cells present with an undifferentiated phenotype and a high tumorigenic potential [26]. The cells were grown in Dulbecco’s modified Eagle’s medium (DMEM) that was supplemented with 10% heat-inactivated fetal bovine serum (FBS), penicillin G (60 mg/L), and streptomycin (100 mg/L) at 37°C in a humidified atmosphere of 5% CO_2_/air. For experimental purposes, the cells were seeded into culture plates or onto glass coverslips.

### Treatment with EGF and Pharmacological Inhibitors

Cell cultures were treated with 20 ng/mL of EGF, a concentration that we have used in a previous study [27]. The effects of EGF treatment on claudin-1 and claudin-3 expression in Caco-2 and HT-29 cells were assessed in cells growing in DMEM medium supplemented with 10% FBS after 6, 24 and 48 h. For pharmacological inhibition, cells were serum starved overnight and selective inhibitors were added to the cell cultures 1 h before EGF treatment to inhibit the intrinsic kinase activity. The cells were then incubated with fresh culture medium supplemented with 10% FBS containing EGF and selective inhibitors, which were maintained throughout the experiments. The inhibitors were diluted in DMSO and stored at −20°C. Each concentrated solution was diluted immediately before each experiment to yield final concentrations of 50 µM (PD98059) and 8 µM (LY294002).

### Plasmid Construction, Production of Recombinant Retroviruses and the Infection of HT-29 Cells

Constructs that contain claudin-1 and claudin-3 murine cDNA have been previously described [28], [29] and were kindly provided by Dr. Mikio Furuse (Division of Cell Biology, Department of Physiology and Cell Biology, Kobe University, Japan). The cDNAs contained in these constructs were subcloned into the pBABE-Puro backbone retroviral plasmid to generate the pBABE-Cld1 (BamH1-Xho1) and pBABE-Cld3 (Bgl2-Xho1) constructs. HEK-293 cells were used as retroviral packaging cells after a transient co-transfection by calcium phosphate precipitation for 24 h with the pCL-Ampho retroviral packaging vector (Cat. no. 10046P; Imgenex, CA, USA) and one of the following constructs: pBABE-Cld1, pBABE-Cld3 or empty retroviral vectors. The cell-free supernatant that contained the virus was collected 48 h after transfection, mixed 1∶1 with fresh medium, supplemented with 8 µg/ml polybrene (Cat. no. 107689; Sigma-Aldrich), and immediately used for the spin-infection (2×45 min at 400×g at room temperature) of 5×10^4^ HT-29 cells. Infected cells were incubated at 37°C for an additional 24 h, trypsinized and used as indicated.

### HT-29^Cld1^ and HT-29^Cld3^ Cell Construction

HT-29^Cld1^, HT-29^Cld3^ or empty-vector (HT-29^pBABE^) cells were generated by transducing HT-29 wild-type cells with pBABE-Cld1, pBABE-Cld3 or empty vectors and selecting successfully transduced cells with 7.5 µg/mL puromycin (Cat. no. P8833; Sigma-Aldrich) for at least 5 days. The clones were isolated and the overexpression of claudins 1 and 3 was confirmed by immunoblotting.

### Claudin-3 Silencing

HT-29 cells were transfected with either a non-targeting control siRNA (siRNA negative control # 1; Cat. no. 4390844; Ambion, TX, USA), scramble as a control for non-sequence-specific effects, or a claudin-3-specific siRNA sequence (Silencer Predesigned siRNA CLDN3, Cat. no. SI03101623; Qiagen, MD, USA). The cells (10^6^) were resuspended in 100 µL of 1SM buffer [30], kindly provided by Dr. Martin Bonamino (INCa, Brazil), containing either the scrambled or CLDN3-specific duplex. The cells were transferred in a 0.2 cm cuvette (Cat. no. MIR 50121; Mirus Biotech, Madison, WI, USA) and electroporated using the W-17 program of the Lonza Nucleofector II electroporation system for HT-29 cells (Lonza Group Ltd, Basel, Switzerland). The cells were then gently resuspended in DMEM supplemented with 10% FBS to a final siRNA concentration of 25 or 45 nM. The attenuation of CLDN3 expression was verified by immunoblotting cell lysates and probing them with an antibody against claudin-3.

### Cell Extraction and Immunoblotting

Caco-2 (3×10^5^) and HT-29 (2×10^5^) cells were seeded into 6-well plates and incubated until confluence. The cell molayers were then scraped and homogenized in lysis buffer (1% Triton X-100, 0.5% sodium deoxycholate, 0.2% SDS, 150 mM NaCl, 2 mM EDTA, 10 mM HEPES, pH 7.3) that contained 20 mM NaF, 1 mM orthovanadate and a protease inhibitor cocktail (1∶100 dilution) to obtain total cell lysates. The homogenized lysates were centrifuged at 10,000×g for 10 min at 4°C. The supernatants were collected and stored at −20°C for later analysis. Equal amounts of protein (30 µg/lane) per sample were separated by SDS-PAGE electrophoresis on 13% gels and transferred to nitrocellulose sheets. The membranes were blocked and incubated with primary antibodies against claudin-1 (1∶500), claudin-3 (2 µg/mL), or α-tubulin (1∶1000). After washing, the membranes were incubated with horseradish peroxidase-conjugated anti-rabbit or anti-mouse antibodies. The proteins were visualized using an enhanced chemiluminescence kit (GE Healthcare, Chalfont St. Giles, UK). The bands were quantified according to their optical densities using LabWorks 4.6 (Bio-Rad Laboratories, Hercules, CA).

### Immunofluorescence

Caco-2 (2×10^5^) and HT-29 (10^5^) cells were seeded on glass coverslips in 24-well plates and incubated until confluence. The cell monolayers were subsequently washed with PBS and fixed with methanol for 10 min at −20°C. Next, the cells were blocked with 0.2% BSA in PBS for 1 h and permeabilized with 0.1% Triton X-100. The cells were incubated overnight with anti-claudin-1, anti-claudin-3 (1∶40), anti-E-cadherin (1∶300) antibodies, followed by 1 h incubations with the appropriate Alexa 488-conjugated secondary antibodies (1∶500). Following the incubations, the cells were mounted using n-propyl-gallate to allow for visualization with either an Axio Observe.Z1 microscope that was equipped with an AxioCam HRc and the AxioVision Release 4.8 digital image processing software (Carl Zeiss Inc., Jena, Germany) or a confocal laser scanning microscope ***FV10i-O***, from which images were analyzed using the FV10-ASW software (Olympus, Tokyo, Japan).

### Wound Healing Assay

HT-29 cells (2×10^5^) were seeded into 6-well plates and incubated until confluence. To perform wound-healing assays, cell monolayers were manually wounded by scraping with pipette tips. After different treatments, the cells were permitted to migrate into the denuded areas and photographed immediately after wounding (0 h) and at 6 h or 24 h after wounding. The distance between the two edges of a denuded area was quantified in triplicate and repeated independently three times. Migration is represented as the percentage of cell migration and plotted on a graph.

### Cell Proliferation Assays

The relative viable cell numbers were determined using crystal violet or trypan blue dyes. The crystal violet method was conducted as described previously [31]. Briefly, the HT-29 cells (10^4^) were seeded into 96-well plates and incubated in culture medium with or without EGF for 24 and 48 h before ethanol fixation for 10 min. A crystal violet solution (0.05% crystal violet and 20% methanol) was added to the cells for 10 min. The cells were washed and solubilized with methanol. The absorbances at 595 nm were measured with a Spectra Max 190 spectrophotometer (Molecular Devices, Sunnyvale, CA, USA). For the trypan blue method, transduced HT-29 cells (2×10^5^) were seeded into 6-well plates and incubated in culture medium for 6 h. After the incubations, the cells were trypsinized (0.05% trypsin/0.02% EDTA in PBS solution) and counted on an Axio Observe.Z1 microscope (Zeiss) with a Neubauer chamber hemacytometer and trypan blue dye (0.4% trypan blue in PBS solution).

### Anchorage-dependent and -independent Colony Formation

For anchorage-dependent colony formation assays, HT-29 cells were seeded at a low density (2.5×10^2^) for 4 h in 12-well plates and treated with EGF for 5 days or left untreated. After 5 days, the cells were fixed with ethanol for 10 min and stained with a crystal violet solution (0.05% crystal violet and 20% methanol). The cells were washed twice with water and solubilized with methanol. The colonies were either counted with an Axio Observer.Z1 microscope (Zeiss) or the absorbances were measured at 595 nm with a Spectra Max190 spectrophotometer (Molecular Devices, Sunnyvale, CA, USA).

For anchorage-independent colony formation assays, 12-well plates were coated with growth medium supplemented with 10% FBS containing 0.6% agarose to avoid cell adhesion to the substrate. HT-29 cells (5×10^2^) were then resuspended in culture medium containing 10% FBS plus 0.3% agarose, seeded on the substrate described above and submitted to different treatments for 11 days (as indicated). At the endpoint, the colonies were counted with an Axio Observer.Z1 microscope (Zeiss).

### Measurement of the Transepithelial Electrical Resistance (TER)

TJ functionality was also assessed by measuring the TER to evaluate the paracellular flux to ions, as previously described by Contreras and colleagues [32] with slight modifications. Briefly, transduced HT-29 cells (10^4^) were seeded at confluence onto Transwell polyester membrane cell culture inserts with a 0.4 µm pore size and 0.33 cm^2^ surface area (Cat. no. CLS3470; Sigma-Aldrich). The TER values were determined using a Millicel-ERS system (Millipore Co., Billirica, MA, USA). The values plotted on the graph were normalized for the area of the insert and the blank value (bath solution incubated with insert without cells) was subtracted. The TER was thus measured in Ohms×cm^2^ (Ω · cm^2^).

### Macromolecule Permeability Assay

To further test the TJ functionality, the macromolecular permeability was assessed using an antibody permeability assay, as described previously [33]. Transduced HT-29 cells (10^5^) were seeded on glass coverslips in 24-well plates and incubated until confluence. The cell monolayers were then fixed in 2% paraformaldehyde and incubated with anti-uvomorulin/E-cadherin antibody for 2 h under nonpermeabilization conditions, followed by 1 h incubation with the respective secondary antibody at 37°C. The uvomorulin/E-cadherin staining was visualized with an Axio Observer.Z1 microscope (Zeiss).

### Statistical Analysis

A statistical analysis was performed with a one-way ANOVA, followed by Dunnett’s post-tests or the t-test using the GraphPad Prism 4.02 software (GraphPad Software, San Diego, CA, USA). The graphs represent the means ± the standard error (SEM) of three independent experiments. A difference of p<0.05 was considered statistically significant.

## Results

### EGF Increases the Protein Levels of Claudin-3 in HT-29 Cells but not in Caco-2 Cells

**Figure 1:**
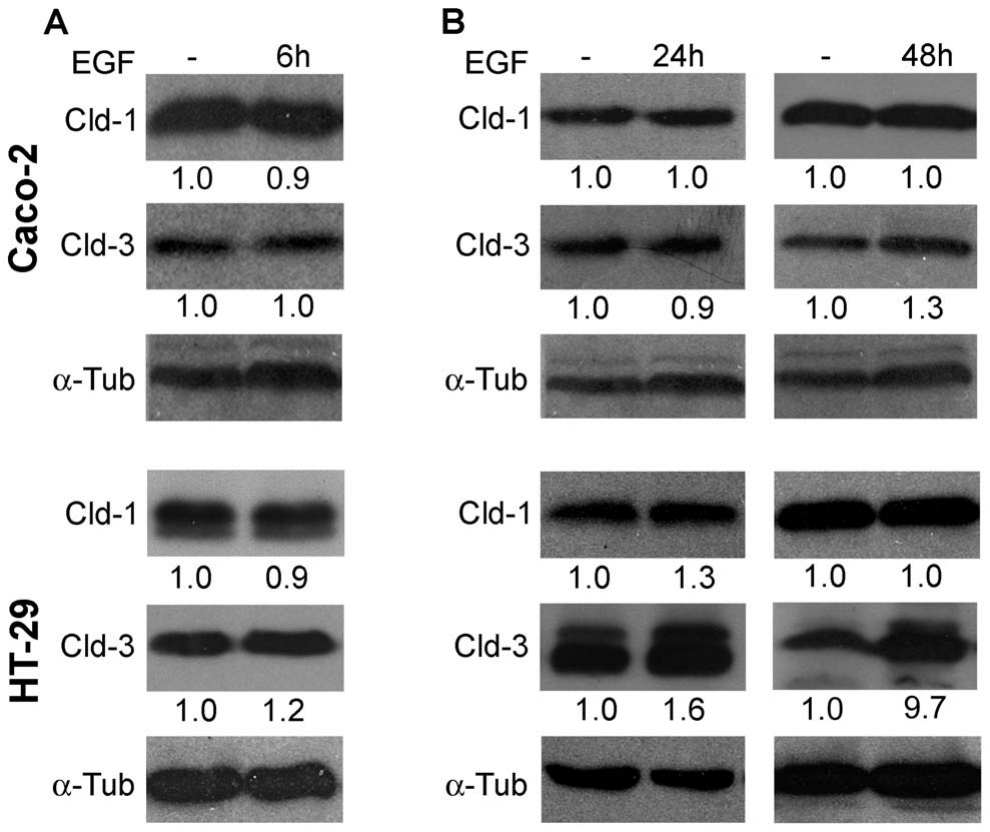
Effect of EGF on the claudins 1 and 3 expression in Caco-2 and HT-29 cells. Cultured Caco-2 and HT-29 cells were treated with EGF (20 ng/mL) for 6 h (**A**), 24 h and 48 h (**B**). Following EGF treatment, total cell lysates were harvested and analyzed by immunoblotting for the expression of claudins 1 and 3. α-tubulin was used as a loading control. The numbers represent the ratio of the optical density of EGF-treated to untreated cells normalized by α-tubulin. *Claudin*; Cld. α*-tubulin*; α-tub.

**Figure 2:**
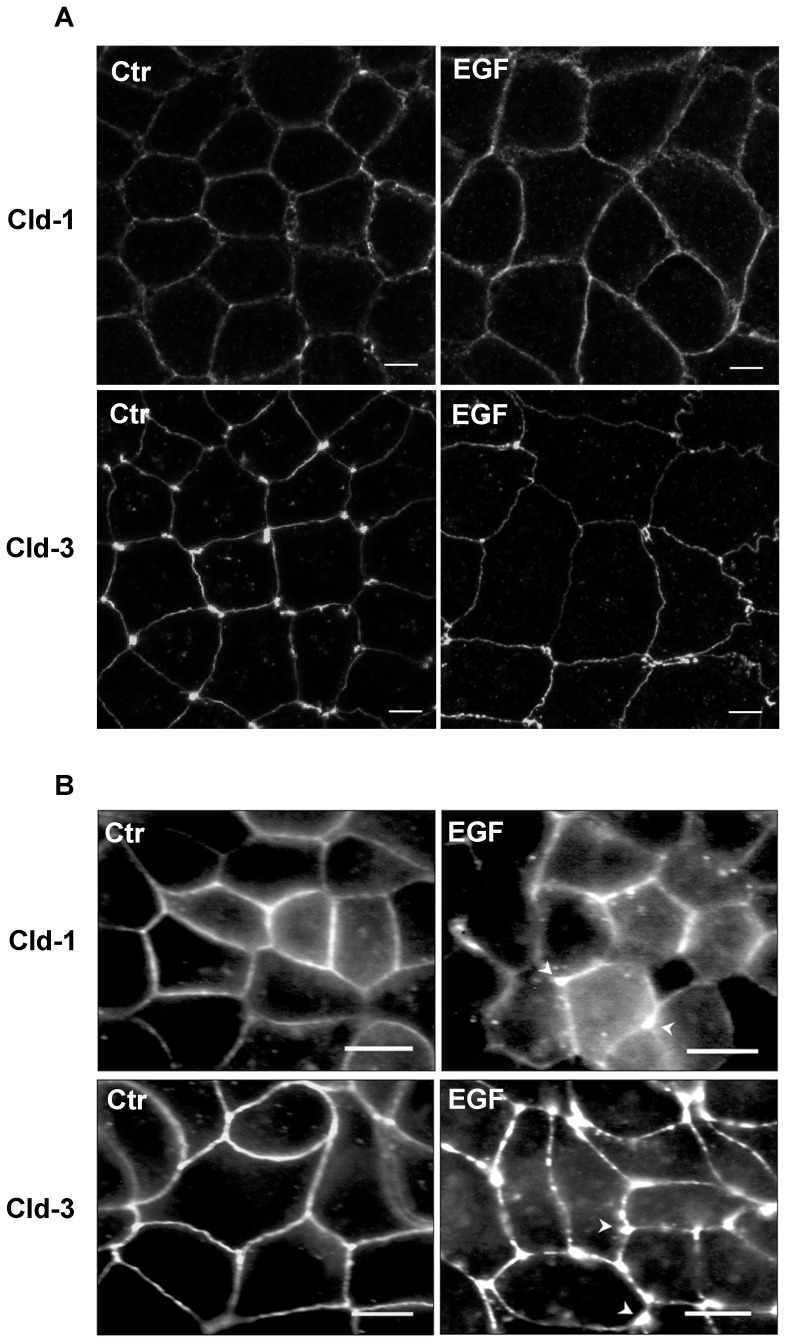
The effect of EGF treatment on the subcellular distribution of claudins 1 and 3. Cell monolayers were grown on glass coverslips and processed for immunofluorescence analysis of claudin-1 and claudin-3 distribution after EGF treatment (48 h). The stained cells were viewed with confocal ***FV10i-O or*** Axio Observe.Z1 microscopes. (**A**) Caco-2 cells***; bar: 5 ***µm. (**B**) HT-29 cells; bar: 10 µm. *Arrowheads*; punctual accumulation of claudins. *Ctr*; control.

Initially, we examined changes in the expression levels of claudin-1 and claudin-3 after EGF treatment in two colorectal adenocarcinoma cell lines, Caco-2 and HT-29, which differ in differentiation status and metastatic potential. We observed that EGF-treatment did not significantly alter the protein levels of claudins 1 and 3 in Caco-2 and HT-29 cells at an early time point (6 h) (Fig. 1A). Following prolonged EGF-treatment times (24 and 48 h), the protein levels of claudins 1 and 3 were not altered significantly in Caco-2 cells. However, HT-29 cells showed significantly increased levels of claudin-3, while the levels of claudin-1 remained unchanged in this same time point of treatment (Fig. 1B). Furthermore, immunofluorescence analysis indicated that EGF treatment for 48 h did not alter the distribution patterns of claudins 1 and 3 in Caco-2 cells (Fig. 2A). Nevertheless, a discontinuous staining pattern and the punctual accumulation of these proteins were observed in some cell-cell contact regions of HT-29 cells (Fig. 2B). Because EGF did not alter the protein levels of claudins 1 and 3 in Caco-2 cells, we examined whether EGF can activate effector pathways in this cell line. We verified that EGF treatment increased the phosphorylation levels of ERK1/2 (Fig. S1), a known signaling effector triggered by EGF. These results indicate that EGF treatment differentially regulates claudins 1 and 3 depending on the cellular context. Based on these results, we chose HT-29 cells for subsequent functional analyses due to the alterations observed in the expression and subcellular distribution of the claudin proteins after EGF treatment.

### EGF Treatment Increases the Migration and Anchorage-Dependent and Anchorage-Independent Colony Formation of HT-29 Cells

**Figure 3:**
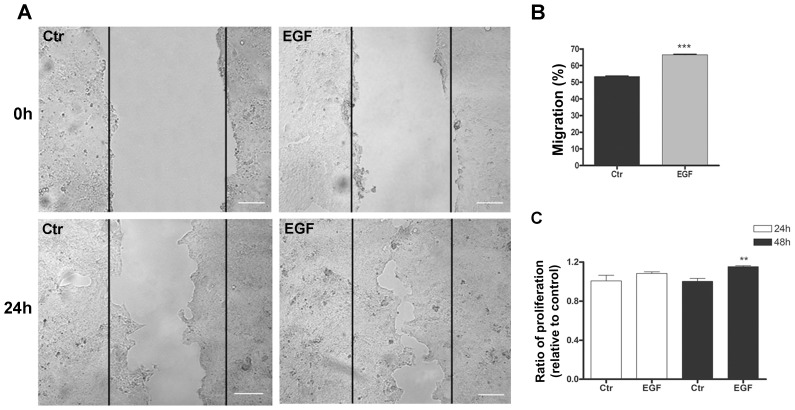
The impact of EGF treatment on the migration and proliferation of HT-29 cells. (**A, B**) HT-29 cells were grown in 6-well plates until confluent. Next, cell monolayers were wounded and treated with EGF, and cell migration in these regions was monitored for 24 h. (**C**) HT-29 cells were seeded in 96-well plates and treated with EGF at the indicated times, and proliferation was quantified using the crystal violet technique. The bar graph shows the ratio of the absorbance of EGF-treated to untreated cells (control). Bar: 100 µm. Error bars indicate the means ± SEM (n = 3); **p<0.01, ***p<0.001 as determined by t test.

We have shown that increased cell migration is related to mechanisms of tumor progression in cancer cells [10], [31] and that EGF treatment increased the migration of Caco-2 cells [34]. To determine whether EGF altered the migration of HT-29 cells, we performed wound-healing assays after EGF treatment. We observed that after 24 h of EGF treatment, the cell migratory potential was increased compared with that of untreated cells (Fig. 3A and 3B). To further investigate whether the wound closure was indeed due to the migration status and not cell proliferation, we verified the relative number of viable cells. Figure 3C shows that cell proliferation was not altered in EGF-treated cells when compared with untreated cells at the same time-point that was observed in the wound-healing assay. An increase in cell proliferation was observed only 48 h after EGF treatment.

**Figure 4:**
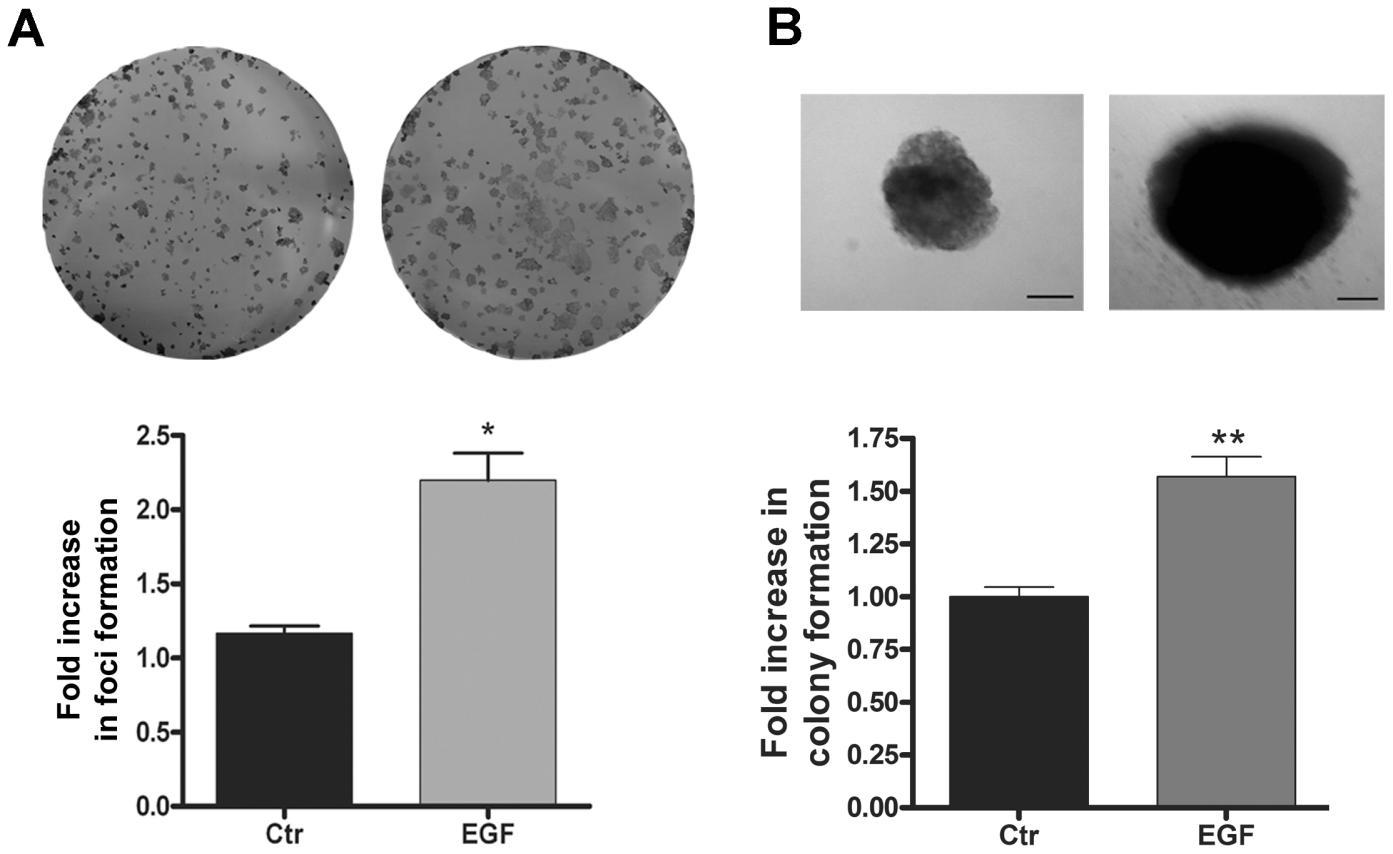
The effect of EGF treatment on anchorage-dependent and anchorage-independent colony formation in HT-29 cells. (**A**) Representative photographs of anchorage-dependent colonies that were stained with crystal violet after EGF treatment. The bar graphs show the ratio of fold increase in foci formation of EGF-treated to untreated cells (control). (**B**) Representative images of anchorage-independent colonies that show the differences in size between EGF-treated and untreated cells. The bar graphs show the ratio of fold increase in colony formation of EGF-treated to untreated cells (control). Bar: 10 µm. Error bars indicate the means ± SEM (n = 3); *p<0.05, **p<0.01 as determined by t test.

It is known that cell transformation is an important parameter to evaluate the malignant potential, which may also be assessed by the ability of cells to form anchorage-dependent and anchorage-independent colonies [35], [36]. In this context, we evaluated the cell-transforming potential of EGF in HT-29 cells. As observed in figure 4A, EGF-treated cells displayed higher numbers of anchorage-dependent colonies when compared with untreated cells. Furthermore, EGF treatment increased the number of anchorage-independent colonies and the colony sizes compared to control cells (Fig. 4B). Together, these data show that EGF enhances events related to the malignant potential of undifferentiated colorectal cancer cells.

### ERK1/2 and PI3K-Akt Signaling Mediate EGF-Induced Increases in Cell Migration and the Colony Formation of HT-29 Cells

**Figure 5:**
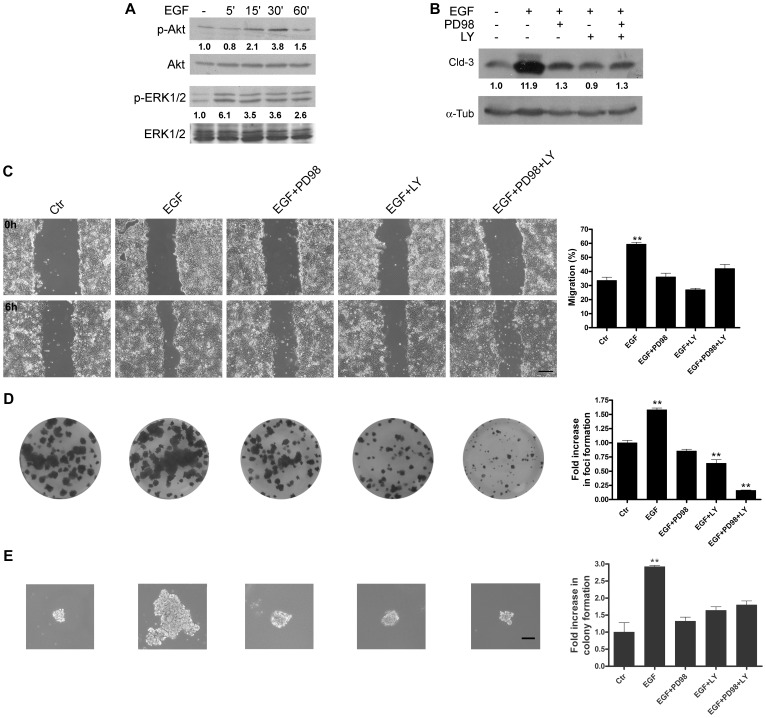
The impact of MEK1/2-ERK1/2 and PI3K-Akt inhibition on EGF-induced effects in HT-29 cells. (**A**) HT-29 cells were grown and treated with EGF for 5, 15, 30 and 60 min, after which the total cell lysates were harvested and analyzed by immunoblotting for p-ERK1/2, ERK1/2, p-Akt, and Akt. (**B**) Cells were grown and pretreated for 1 h with the indicated inhibitors before incubation with EGF for 48 h. Total cell lysates were harvested and analyzed by immunoblotting for claudin-3. α-Tubulin was used as a loading control. The numbers represent the ratio of the optical density of treated to untreated cells normalized by total protein or α-tubulin. Representative images of wound healing (**C**), anchorage-dependent colony formation (**D**) and anchorage-independent colony formation (**E**) assays. The cells were pretreated with the inhibitors as indicated, and the assays were performed as described in the Materials and Methods. For the anchorage-dependent assay, the bar graph shows the ratio of the fold increase in foci formation of treated to untreated cells (control). For the anchorage-independent assay, the bar graphs show the ratio of the fold increase in colony formation of treated to untreated cells (control). Bar: 200 µm. Error bars indicate the means ± SEM (n = 3); **p<0.01 as determined by an ANOVA.

EGF is well known to activate signaling pathways, such as ERK1/2 and PI3K-Akt, to regulate cell proliferation, survival and differentiation in various tumor cell models. Therefore, we analyzed whether these pathways could also modulate the underlying EGF-induced events that we had observed here, including the increase in claudin-3 expression. As seen in figure 5A, EGF treatment increased the phosphorylation levels of ERK1/2 and Akt, indicating the activation of these proteins. Next, we pharmacologically inhibited the MEK1/2-ERK1/2 and PI3K-Akt pathways with PD98059 and LY294002, respectively, to verify whether these pathways could modulate the EGF-induced increase in claudin-3 expression levels in HT-29 cells. We observed that treatment with these inhibitors alone or in combination prevented the EGF-induced increase in claudin-3 expression levels (Fig. 5B). Furthermore, we observed that inhibition of the ERK1/2 and PI3K-Akt signaling pathways prevented the EGF-induced increase in cell migration (Fig. 5C). In figures 5D and 5E, we further verified that the inhibition of both pathways prevented EGF-induced increases in the numbers of anchorage-dependent and anchorage-independent colonies. Interestingly, pretreatment with the PI3k inhibitor alone and in combination with the ERK1/2 inhibitor decreased the anchorage-dependent colony formation. Together, these data strongly support the notion that the ERK1/2 and PI3K-Akt pathways are also involved in increased claudin-3 expression levels, concomitant to the increased malignant potential that is induced by EGF in HT-29 cells.

### Forced Overexpression of Claudin-1 and Claudin-3 increases the TER, but Claudin-3 Overexpression Facilitates the Paracellular Flux to Macromolecules

**Figure 6:**
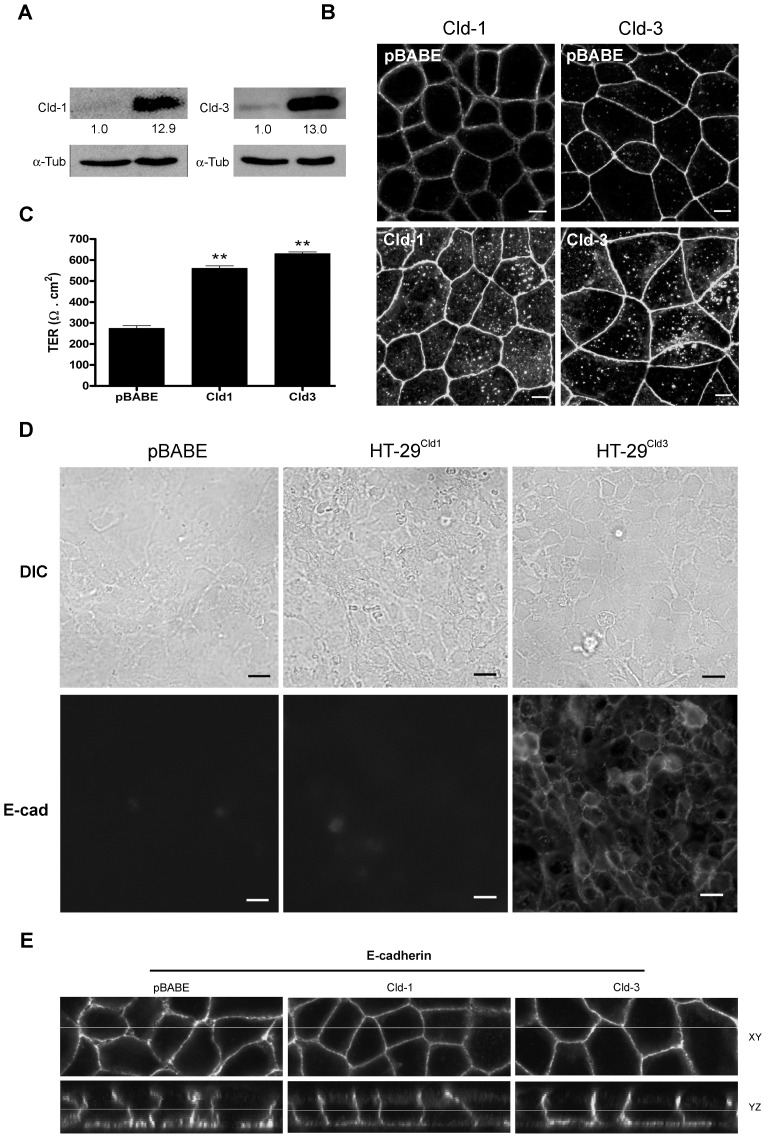
Effects of the forced expression of claudins 1 and 3 on their subcellular distribution, TER and macromolecular permeability. HT-29 cells were transduced with retroviral vectors that contained claudin-1, claudin-3, or empty vector (pBABE). (**A**) The cells were grown and total cell lysates were harvested and analyzed by immunoblotting for the expression of claudin-1 and claudin-3. The numbers represent the ratio of the optical density of claudin-transduced to empty vector-transduced cells normalized by α-tubulin. (**B**) Cells were grown on glass coverslips and processed under permeabilizing conditions for the immunofluorescence analysis of claudin-1 and claudin-3 distribution; bar: 5 µm. (**C**) Cells were grown on Transwell inserts and the TER was measured using the Millicel-ERS system. The bar graphs show the TER values normalized for the area of the insert with the blank value subtracted. (**D**) The cells were grown on glass coverslips and processed for immunofluorescence under non-permeabilizing conditions to evaluate macromolecular permeability using the anti-uvomorulin/E-cadherin antibody; bar: 10 µm. (**E**) Cells were grown on glass coverslips and processed under permeabilizing conditions for immunofluorescence analysis of E-cadherin distribution. The stained cells were viewed with ***FV10i-O*** confocal microscope. Error bars indicate the means ± SEM (n = 3); **p<0.01 as determined by an ANOVA.

Based on the results described above, we postulated that the differential expression levels of claudin-1 and claudin-3 play important roles in colorectal cancer progression. To further test this hypothesis, we forced the expression of claudin-1 and claudin-3 cDNA in HT-29 cells, and the resulting cells were named HT-29^Cld-1^ and HT-29^Cld-3^, respectively. Immunoblot analysis confirmed robust claudin-1 (HT-29^Cld-1^) and claudin-3 (HT-29^Cld-3^) overexpression compared with cells that were transduced with the empty vector (HT-29^pBABE^) (Fig. 6A). Furthermore, cells that overexpressed these proteins displayed increased cytoplasmic staining; however, the labeling was maintained at cell-cell contacts (Fig. 6B).

In a previous study, we showed that the upregulation of claudins 1, 3 and 4 was associated with the disorganization of TJ fibrils, leading to the increased permeability of the paracellular barrier in tissue samples of human colorectal cancer [19]. Accordingly, we measured the TER to evaluate the effects of claudin-1 and claudin-3 overexpression on the paracellular flux to ions. As observed in figure 6C, claudin-1 and claudin-3 overexpression increased the TER of HT-29 cells, indicating a strengthening of the barrier to ions. It is also known that the rearrangement of TJ strands may favor the paracellular flux of macromolecules, thus impairing the barrier function of TJs [37]. We and other authors have assessed the TJ function using an antibody permeability assay, which also evaluates the paracellular permeability to macromolecules [27], [33]. Using this assay, we observed an absence of uvomorulin/E-cadherin staining in HT-29^cld-1^ cells, which indicated that cells overexpressing claudin-1 maintained an intact TJs-regulated paracellular flux of macromolecules. On the contrary, cells that overexpressed claudin-3 (HT-29^Cld-3^) showed a normal pattern of uvomorulin/E-cadherin staining on the plasma membrane, which indicated that the macromolecular flux, was impaired because the cells were not permeabilized prior to staining (Fig. 6D). To confirm that this labeling pattern did not result from the claudin overexpression-induced cellular redistribution of E-cadherin, we assessed the distribution of this protein in permeabilized cells by immunofluorescence and confocal microscopy. We observed that claudin-1 and claudin-3 overexpression did not alter the cellular distribution of the E-cadherin, which was mostly present in the basolateral region (Fig. 6E). Together, these data indicate that claudin-1 and claudin-3 overexpression increases the TER, but claudin-3 overexpression facilitates the macromolecular flux in HT-29 cells.

### Forced Overexpression of Claudin-3 but not of Claudin-1 Increases the Malignant Potential of HT-29 Cells

**Figure 7:**
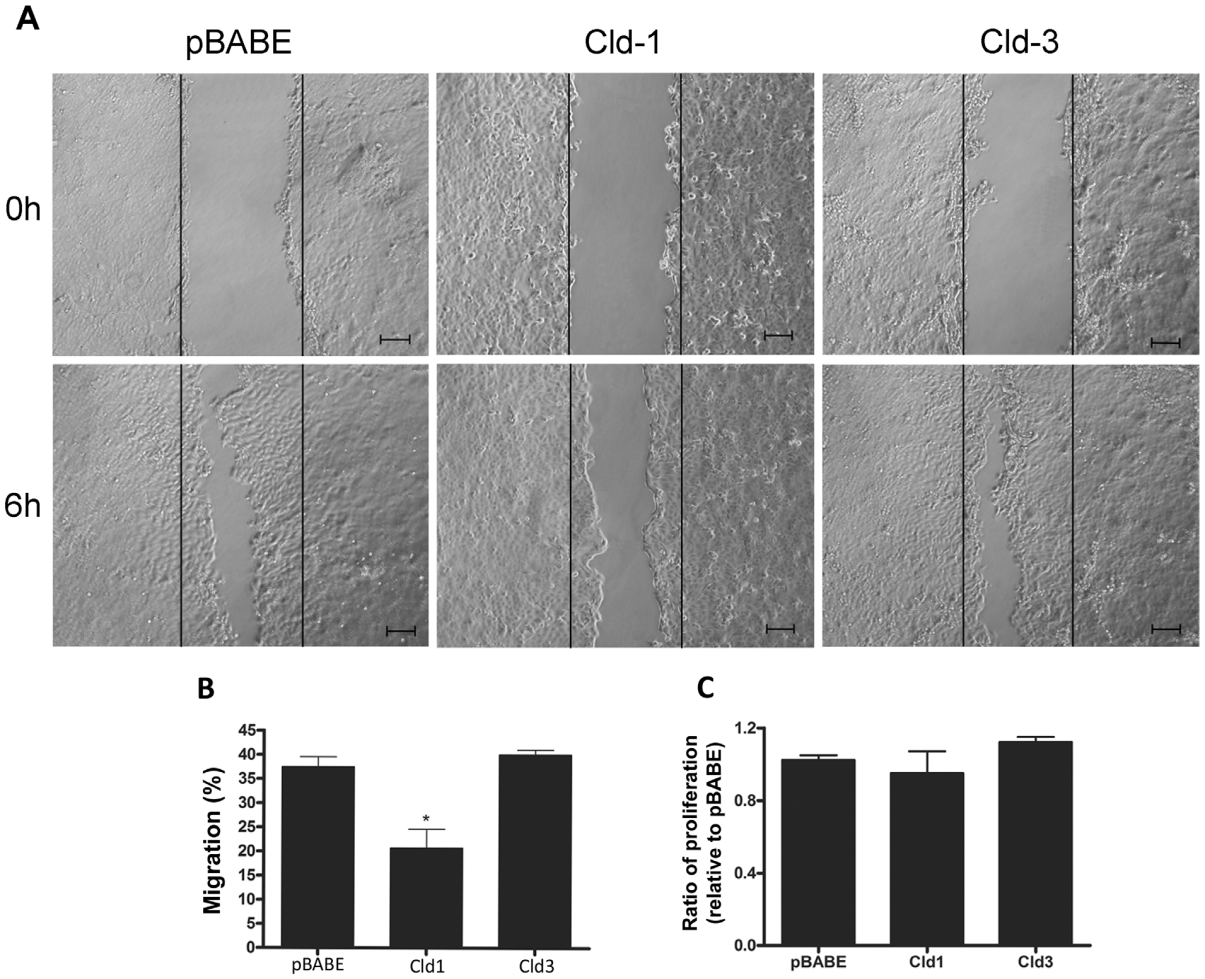
The effect of claudin-1 and claudin-3 overexpression on migration and proliferation. (**A, B**) HT-29^pBABE^, HT-29^cld-1^ and HT-29^cld-3^ cells were grown in 6-well plates until confluent. Next, cell monolayers were wounded and the cell migration in the wounded regions were monitored after 6 h. (**C**) Transduced cells were seeded into 6-well plates for 6 h, and the numbers of cells were quantified by optical microscopy using trypan blue dye as described in the Materials and Methods. The bar graph shows the ratio of the number of claudin-transduced to the empty vector-transduced (pBABE) cells. Bar: 100 µm. Error bars indicate the means ± SEM (n = 3); *p<0.05 as determined by an ANOVA.

**Figure 8:**
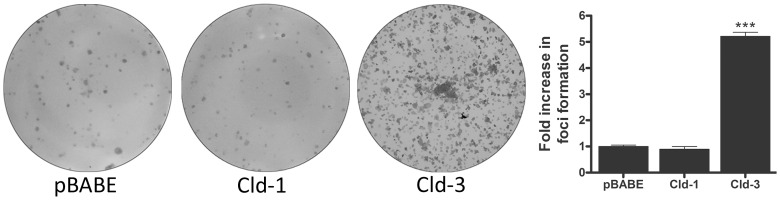
The impact of claudin-1 and claudin-3 overexpression on anchorage-dependent colony formation. Representative photographs of anchorage-dependent colonies of transduced HT-29 cells (HT-29^pBABE^, HT-29^cld-1^ and HT-29^cld-3^) that were stained with crystal violet. The bar graph shows the ratio of the fold increase in foci formation of claudin-transduced to empty vector-transduced (pBABE) cells. Error bars indicate the means ± SEM (n = 3); ***p<0.001, as determined by an ANOVA.

**Figure 9:**
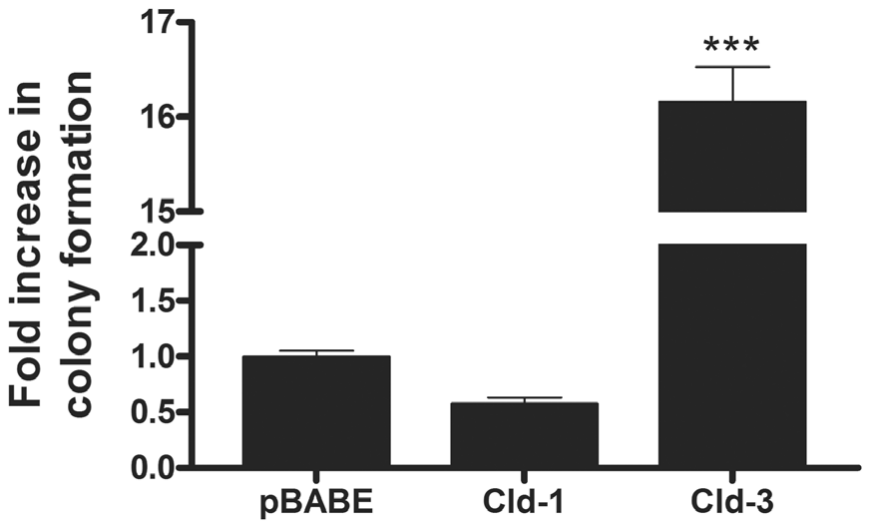
The impact of claudin-1 and claudin-3 overexpression on anchorage-independent colony formation. HT-29^pBABE^, HT-29^cld-1^ and HT-29^cld-3^ cells were seeded for an anchorage-independent colony formation assay as described in the Materials and Methods. The bar graph shows the ratio of the fold increase in colony formation of claudin-transduced cells to empty vector-transduced (pBABE) cells. Error bars indicate the means ± SEM (n = 3); ***p<0.001, as determined by an ANOVA.

We observed that EGF treatment did not cause statistically significant changes in claudin-1 expression but increased claudin-3 expression concomitantly with the malignant potential in HT-29 cells (Fig. 1B, 3, 4 and 5). Based on these findings, we further examined the effects of the forced claudin-1 and claudin-3 overexpression on cell migration, proliferation and colony formation. Interestingly, we observed that the overexpression of claudin-1 but not that of claudin-3 decreased cell migration (Fig. 7A and 7B). Conversely, cell proliferation was not altered by the overexpression of either claudin at the same time point (Fig. 7C). Furthermore, we observed increases in the anchorage-dependent and anchorage-independent colonies formation after the overexpression of claudin-3 but not claudin-1 (Fig. 8 and 9, respectively). Because claudin-3 overexpression increased the colony formation in HT-29 cells, we assessed whether the forced overexpression of claudin-3 could increase cell proliferation at later time points (24, 48 and 72 h). We verified that claudin-3 overexpression increased cell proliferation at these times (Fig. S2), which could contribute to the increased malignant potential of HT-29 cells. These results indicate that the differential expression of claudins 1 and 3 plays a crucial role in the malignant phenotype of the HT-29 colorectal cancer cells.

### Claudin-3 Silencing Prevents the EGF-induced Malignant Potential of HT-29 Cells

**Figure 10:**
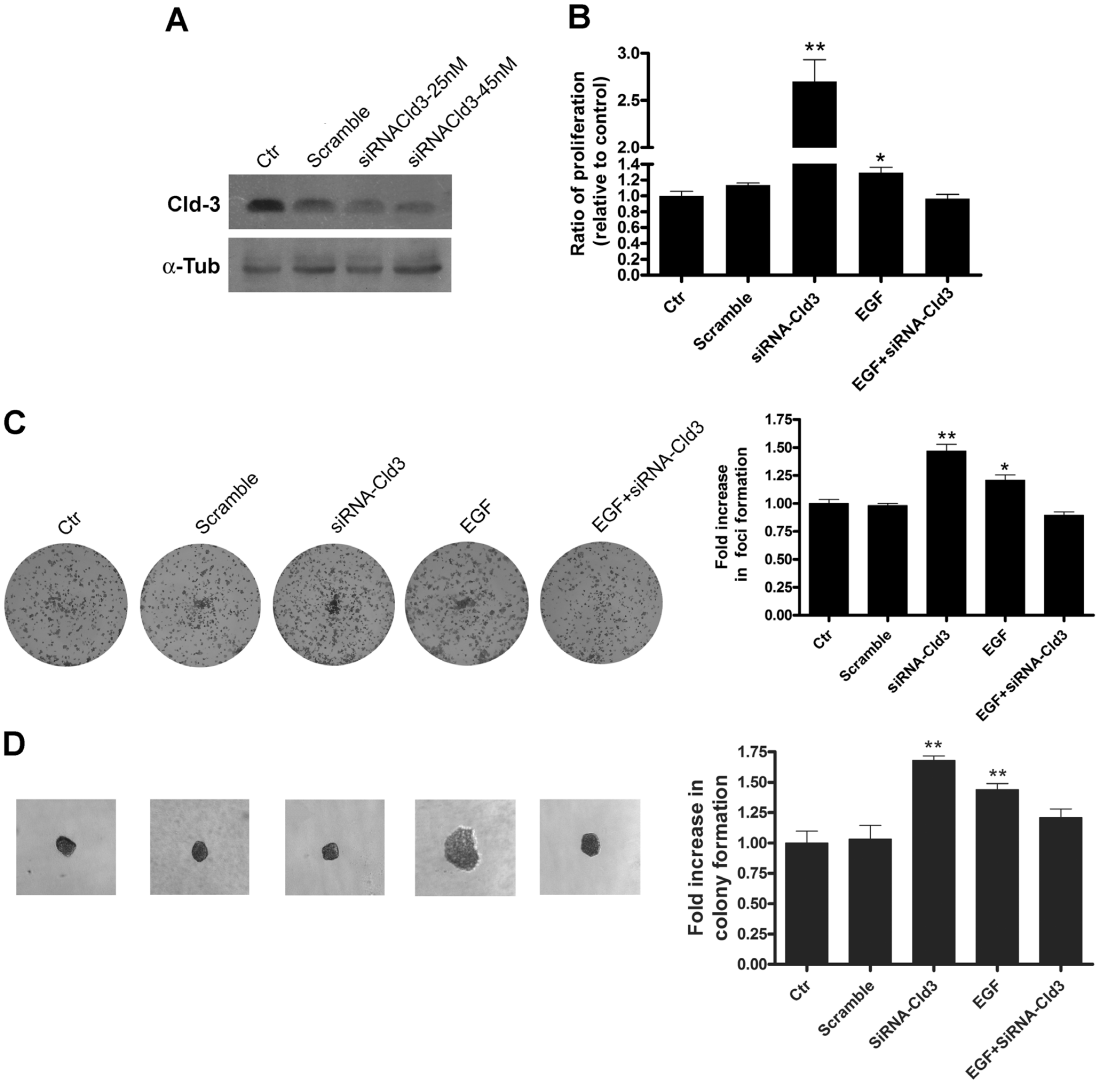
The impact of claudin-3 silencing on EGF-induced effects in HT-29 cells. HT-29 cells were transfected with non-targenting control siRNA (scramble) or claudin-3 siRNA and treated with EGF as indicated. (**A**) Cells were grown for 24 h and the total cell lysates were obtained and analyzed for the expression of claudin-3 by immunoblotting; α-tubulin was used as a loading control. (**B**) Cells were seeded in 96-well plates for 48 h and proliferation was quantified using the crystal violet technique. The bar graph shows the ratio of the absorbance of transfected and/or EGF-treated cells to the control cells. (**C**) Representative photographs of anchorage-dependent colonies that were stained with crystal violet. The bar graph shows the proportion of fold increase in foci formation of the transfected and/or EGF-treated cells to control cells. (**D**) Representative images of anchorage-independent colonies. The bar graph shows the ratio of the fold increase in colony formation of transfected and/or EGF-treated cells to control cells. Error bars indicate the means ± SEM (n = 3); *p<0.05, **p<0.01, as determined by an ANOVA. *Claudin*; Cld. α*-tubulin*; α-tub.

Because EGF treatment increased claudin-3 expression (Fig. 1B), and the overexpression of this claudin is related to the increased malignant potential of HT-29 cells (Figs. 8 and 9), we investigated whether the downregulation of claudin-3 could to prevent the EGF-induced effects in HT-29 cells using claudin-3 siRNA. Using immunoblot analysis, we confirmed a robust downregulation of claudin-3 24 h after transfection using both 25 and 45 nM of claudin-3 siRNA (Fig. 10A). Based on these results, we chose 25 nM of claudin-3 siRNA for subsequent functional analyses. Because claudin-3 overexpression-regulated colony formation is analyzed at late time points (more than 5 days), we evaluated whether the early downregulation of claudin-3 induced by siRNA (at 24 h) would be sufficient to prevent the events regulated by prolonged treatment with EGF. Accordingly, we performed a proliferation assay after 48 h of treatment with EGF because we had observed that this agent increased cell proliferation at this time point (Fig. 3C). We observed that claudin-3 siRNA prevented the increase in EGF-induced proliferation, indicating that the early downregulation of claudin-3 may regulate EGF-induced late events (Fig. 10B). In addition, claudin-3 siRNA also prevented the increase in EGF-induced anchorage-dependent and -independent colony formation (Figs. 10C and D, respectively). Interestingly, a higher proliferation rate and more colony formation were observed using only the claudin-3 siRNA. Together, these data indicate that claudin-3 downregulation alone increases the malignant potential but may also prevent the EGF-induced malignant potential of HT-29 cells. These findings suggest that an imbalance in the levels of this claudin plays an important role in the development of the tumorigenic process.

## Discussion

Changes in the expression and subcellular distribution of claudin proteins have been reported in different epithelial cancers in which EGF signaling is involved. However, the roles that EGF plays in modulating the expression of these proteins are different and depend of the cell type and tumor. In a previous study using tissue samples of patients with colorectal cancer, we showed the increased expression of claudins 1, 3 and 4, which was associated with a significant disorganization of the TJ strands and increased paracellular permeability [19]. However, the molecular mechanisms involved in the regulation of this overexpression and its implications during the progression of this cancer type remained undefined. In the present study, we demonstrated that EGF increased the expression of claudin-3 protein, an event that was mediated by the ERK1/2 and PI3K-Akt pathways. We further demonstrated that the increased expression of claudin-1 and claudin-3 differentially affected colorectal cancer cells; in HT-29 cells, the forced expression of claudin-1 decreased cell migration, whereas the forced expression of claudin-3 increased the malignant potential. Moreover, claudin-3 silencing prevented the EGF-induced increase in the malignant potential of HT-29 cells.

Our study reveals that EGF treatment did not alter the protein levels of claudin-1 and claudin-3 in Caco-2 cells; however, HT-29 cells treated with this agent displayed differential changes in the expression of these proteins. Claudin-1 levels were not significantly altered after of 6, 24 or 48 h, while the expression levels of claudin-3 did not change significantly at 6 h of treatment but were increased at 24 and 48 h, and the increase was more evident in this later time (Fig. 1A and 1B). The differentiation grades and metastatic behaviors of the cell lines used in the study may explain these results. Claudin-2 overexpression has been shown to relate to epithelial weakening in lung and colorectal cancer cells, and these events are regulated by EGF-mediated signaling pathways [18], [22]. Here, we observed changes in the redistribution of claudins 1 and 3 after prolonged EGF treatment in HT-29 cells but not in Caco-2 cells (Fig. 2A and 2B), even though EGF signaling was triggered in this cell line as shown by increased ERK1/2 phosphorylation (Fig. S1). The EGF-induced changes observed in this study could be attributed to impaired TJ function, which facilitated access to the EGF receptor, EGFR. This receptor is mainly present in the basolateral region and can thus potentially induce neoplastic transformation, as suggested previously [14], [19], [38]. Controversially to our observations, a recent study showed that EGF decreased claudin-3 and -4 via MEK/ERK and/or PI3K/Akt signaling pathways in ovarian cancer cell lines [39]. The reasons for the discrepancy observed are currently unclear but may be related to tissue-specific differences in claudin function or even the tissue microenvironment features, as previously discussed [40].

In earlier studies, we showed a correlation between increased cell migration and malignant potential in colorectal cancer cells [10], [31], [34]. Here, we showed increased cell migration in HT-29 cells that were treated with EGF (Fig. 3A and 3B), which confirms that EGF is a potential tumor promoter. Furthermore, we observed that inhibitors of the ERK1/2 and PI3K-Akt pathways both prevented and decreased the EGF treatment-induced migration of HT-29 cells (Fig. 5C), suggesting that these pathways participate as modulators of EGF-mediated responses. In addition, to verify the importance of claudins 1 and 3 in this process, we forced the overexpression of these proteins in HT-29 cells. Compared to HT-29^pBABE^ cells, our results showed that HT-29^Cld-1^ cells displayed decreased cell migration, whereas the migration of HT-29^Cld-3^ cells was unaltered (Fig. 7A and 7B). These results suggest that claudin-1 expression plays an important role in cell migration and that this migration decreases with claudin-1 upregulation. Conversely, overexpressed claudin-3 *per se* was not sufficient to alter cell migration. It is possible that EGF may induce other pathways that are involved in the regulation of cell migration; however, further studies are necessary to confirm this hypothesis.

We further evaluated the malignant potential of EGF-treated HT-29 cells with colony formation assays [35], [41] and observed that EGF treatment increased the numbers of anchorage-dependent and anchorage-independent colonies (Fig. 4A and 4B). Additionally, the inhibition of PI3K-Akt and ERK1/2, alone or in combination prevented these effects induced by EGF (Fig. 5D and 5E). Furthermore, we showed that forced claudin-3 overexpression increased colony formation, while claudin-1 overexpression did not alter the colony formation potential (Fig. 8 and 9). These findings agree with previous reports, which showed that the upregulation of claudins 3 and 4 was related to increased tumorigenic potential in ovarian epithelial cells [42], [43]. Nevertheless, our findings show that claudin-1-overexpressing cells displayed decreased malignant potential, as determined by the prevention of cell migration, and this finding is in contrast with previously published reports in which claudin-1 upregulation increased metastatic potential in colorectal cancer [44], [45]. We do not know the reason for these different observations, but they may be a result of cell type differences; we used HT-29 cells, which are less differentiated than the SW480 and SW620 cells used by the previous authors [46]. Additionally, earlier studies have shown that claudin-1 downregulation was related to disease recurrence and poor patient survival in stage II colon cancer [47] and that the decreased expression of this protein was also observed in lymph node metastases of colon carcinomas. We postulate that claudin-1 expression and colon cancer progression correlate negatively, as previously suggested [21]. Based on our findings, we suspect that the differential regulation of claudins 1 and 3 expression may define a more aggressive phenotype in colorectal cancer cells. Currently, we are examining the differential regulation of these claudins in colorectal cancer in detail.

We also showed that the forced overexpression of claudin-1 and claudin-3 increased the cytoplasmic content of these proteins but did not alter their localization at cell-cell contacts (Fig. 6B). Moreover, we observed that claudin-1 and claudin-3 overexpression increased the TER, indicating a higher retention of the paracellular flux to ions (Fig. 6C). However, the HT-29 cells that overexpressed claudin-3 increased the macromolecular permeability, indicating a higher paracellular flux to macromolecules (Fig. 6D). It is known that the paracellular flux is performed differentially, depending on the type of claudin and is cell specific. For instance, upregulation of claudin-3 decreased the barrier function, while the overexpression of claudin-4 improved the alveolar TER [48]. On the other hand, H_2_O_2_-induced downregulation of claudin-3 decreased the TER and increased the FITC-dextran permeability in gastric epithelial cells [49]. Additionally, claudin-2 was suggested have a role in the regulation of ion-selective passage, while other TJ proteins, such as occludin and tricellulin, appear to regulate the macromolecular flux [50]. In our study, we verified that claudin-3 overexpression did not alter the E-cadherin subcellular redistribution, indicating that the altered macromolecular permeability, as assessed by the labeling of Uvomorulin/E-cadherin, is a consequence of impaired TJ function and not the redistribution of this protein (Fig. 6E). These findings indicate that claudin-3 overexpression regulates of dual manner the barrier function of TJs, strengthening the barrier to ions while facilitating the macromolecular flux, which may be a reflex of the redistribution of occludin e/or tricellulin to the cytoplasm. However, further studies are necessary to better understand the role played by interactions between claudins and other TJ proteins to regulate TJ barrier function.

We further investigated the importance of claudin-3 as regulator of EGF-induced events in HT-29 cells by using siRNA for this claudin. We showed that the silencing of claudin-3 prevents the EGF-induced cell proliferation increases and anchorage-dependent and -independent colony formation (Fig. 10B, 10C and 10D). Interestingly, we verified that claudin-3 silencing alone caused increased cell proliferation and colony formation more than EGF treatment. Based these findings, it is possible suggest that both claudin-3 overexpression and claudin-3 downregulation may cause the disorganization of TJs, which could contribute to the malignant potential of these cells. In addition, our data indicate that equilibrium of the claudin-3 level maintains the homeostasis of the intestinal epithelium and that both claudin-3 upregulation or downregulation may result in increased malignant potential. However, additional experiments are needed to confirm this mechanism.

In conclusion, we have shown that claudin-3 overexpression is associated with the increased malignant potential of HT-29 cells and that the ERK1/2 and PI3K-Akt pathways, activated by EGF, are important regulators of this event. Additionally, the impaired paracellular flux of macromolecules in HT-29 cells that overexpress claudin-3 confirms the correlation between impaired epithelial barrier function and colorectal tumorigenesis. Furthermore, we showed that claudin-3 silencing can prevent the EGF-induced increase of the malignant potential. However claudin-3 silencing alone can enhance this event, indicating a fine balance that controls the expression of this claudin to maintain intestinal homeostasis. Most importantly, our study contributes to a better understanding of the molecular mechanisms that regulate the expression of claudins and the relationship of these proteins with the malignant process. Lastly, our study indicates possible molecular targets for future applications in the treatment of colorectal cancer.

## Supporting Information

Figure S1 **Effect of EGF on the activation of ERK1/2 proteins in Caco-2 cells.** Cells were grown and treated with EGF for 15 and 30 min, 1, 2 and 6 h, after which total cell lysates were harvested and analyzed by immunoblotting for p-ERK1/2 and ERK1/2. The numbers represent the ratio of optical density of the pERK1/2 of EGF-treated to untreated cells normalized by ERΚ1/2.
(TIF)

Figure S2 **Effect of claudin-3 overexpression on proliferation for prolonged times.** Transduced cells were seeded into 6-well plates, and the numbers of cells were quantified by optical microscopy after 24, 48 or 72 h using trypan blue dye as described in the Materials and Methods. The bar graph shows the ratio of the number of claudin-transduced cells to empty vector-transduced (pBABE) cells. Error bars indicate the means ± SEM (n = 3); **p<0.01 as determined by a t-test.
(TIFF)
