# Supplementary figures and images for: Claudin-3 Overexpression Increases the Malignant Potential of Colorectal Cancer Cells: Roles of ERK1/2 and PI3K-Akt as Modulators of EGFR signaling
Source: PLoS One. 2013 Sep 19;8(9):e74994. doi: 10.1371/journal.pone.0074994 (PMC3777902; doi:10.1371/journal.pone.0074994)

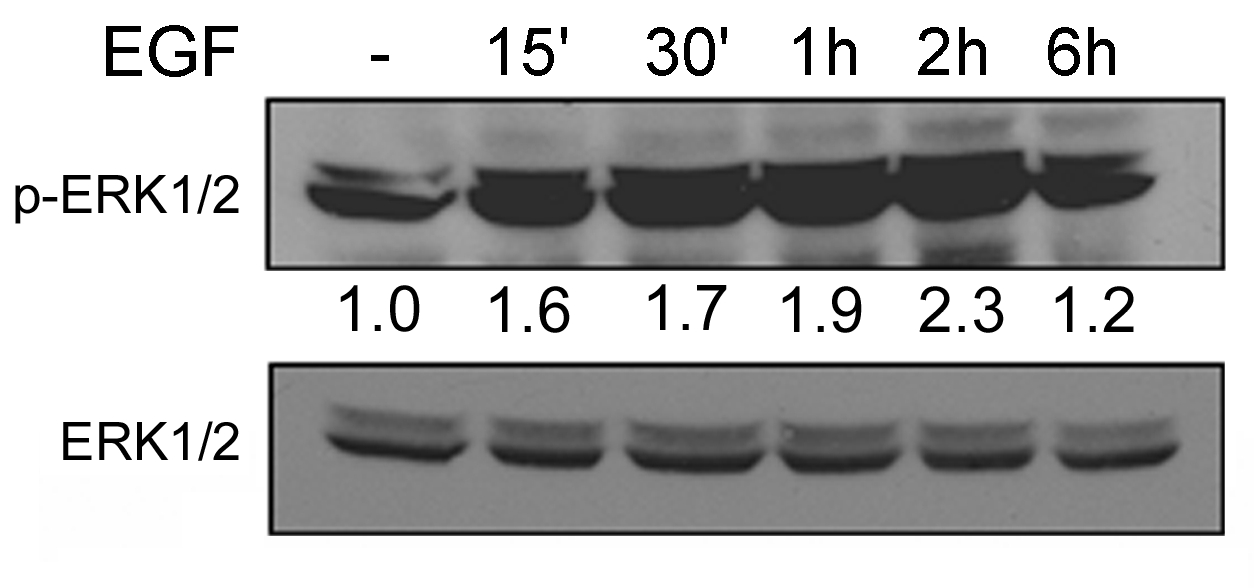

Supplement: Figure S1 — Effect of EGF on the activation of ERK1/2 proteins in Caco-2 cells. Cells were grown and treated with EGF for 15 and 30 min, 1, 2 and 6 h, after which total cell lysates were harvested and analyzed by immunoblotting for p-ERK1/2 and ERK1/2. The numbers represent the ratio of optical density of the pERK1/2 of EGF-treated to untreated cells normalized by ERΚ1/2. (TIF) [file pone.0074994.s001.tif]

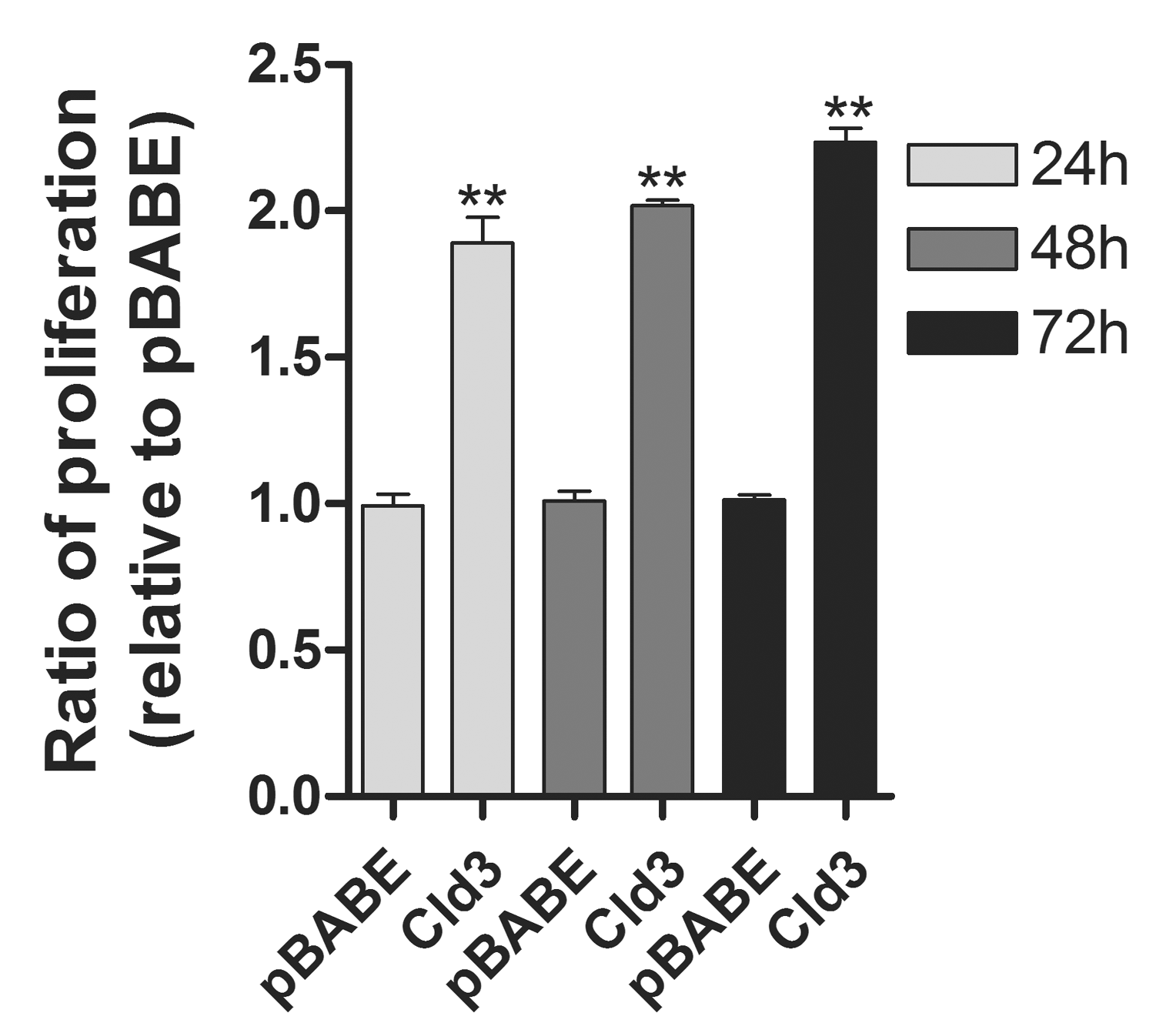

Supplement: Figure S2 — Effect of claudin-3 overexpression on proliferation for prolonged times. Transduced cells were seeded into 6-well plates, and the numbers of cells were quantified by optical microscopy after 24, 48 or 72 h using trypan blue dye as described in the Materials and Methods. The bar graph shows the ratio of the number of claudin-transduced cells to empty vector-transduced (pBABE) cells. Error bars indicate the means ± SEM (n = 3); **p<0.01 as determined by a t-test. (TIFF) [file pone.0074994.s002.tiff]
